# General-purpose pre-trained large cellular models for single-cell transcriptomics

**DOI:** 10.1093/nsr/nwae340

**Published:** 2024-09-25

**Authors:** Haiyang Bian, Yixin Chen, Erpai Luo, Xinze Wu, Minsheng Hao, Lei Wei, Xuegong Zhang

**Affiliations:** MOE Key Laboratory of Bioinformatics and Bioinformatics Division of BNRIST, Department of Automation, Tsinghua University, China; MOE Key Laboratory of Bioinformatics and Bioinformatics Division of BNRIST, Department of Automation, Tsinghua University, China; MOE Key Laboratory of Bioinformatics and Bioinformatics Division of BNRIST, Department of Automation, Tsinghua University, China; MOE Key Laboratory of Bioinformatics and Bioinformatics Division of BNRIST, Department of Automation, Tsinghua University, China; MOE Key Laboratory of Bioinformatics and Bioinformatics Division of BNRIST, Department of Automation, Tsinghua University, China; MOE Key Laboratory of Bioinformatics and Bioinformatics Division of BNRIST, Department of Automation, Tsinghua University, China; MOE Key Laboratory of Bioinformatics and Bioinformatics Division of BNRIST, Department of Automation, Tsinghua University, China; Center for Synthetic and Systems Biology, School of Life Sciences and School of Medicine, Tsinghua University, China

The great capability of AI large language models (LLMs) pre-trained on massive natural language data has inspired scientists to develop a few large-scale AI foundation models for single-cell transcriptomics, or large cellular models (LCMs). LCMs are first pre-trained on massive single-cell RNA-seq data in a self-supervised manner without specific design for downstream tasks. Then, through transfer learning and model fine-tuning, they have demonstrated superior performance across a wide spectrum of tasks such as cell type annotation, data integration, and drug-sensitivity or perturbation response prediction. The success opened a promising new route toward developing AI models to grasp underlying biological knowledge from massive data to a scale that cannot be handled by human analysis. This review introduces the basic principles, major technical variations, and typical applications of current LCMs, and shares our perspective on open questions and future directions of this exciting field.

High-throughput single-cell sequencing technologies have enabled the accumulation of massive transcriptome data on human cells, covering hundreds to thousands of cell types and various physiological or pathological states. There have been many efforts to collect, archive or assemble the data, such as the Human Cell Atlas (HCA) [1], the Human Ensembled Cell Atlas (hECA) [2] and CZ-CELLxGENE [3]. The accumulation of data and the development of many single-cell bioinformatic methods have advanced many fields of biological and medical studies. Most advances are discoveries that pertain to specific biological questions. Some scientists have also begun to study general questions about how to organize the massive data as a unified system, and how to build generic AI models to learn the biology underneath the data [4]. The success of pretrained foundation models in natural language processing (NLP), computer vision (CV) and other fields has inspired researchers to develop AI foundation models for single-cell transcriptomics that can learn the inherent ‘semantics’ and ‘grammar’ of the gene expression language of cells from massive single-cell data [5–11].

‘Foundation model’ in AI refers to machine-learning models that can be adapted for a wide range of downstream tasks after being pretrained on large-scale data through extensive self-supervised learning. Foundational models were first used in the NLP field, where they are often called large language models (LLMs) as the models are very large. The Transformer structure [12] is currently the most powerful backbone architecture for LLMs and foundation models in other fields. It is a deep neural network model with many stacks of multilayer perceptrons (MLPs) and multi-head self-attention modules that enable the learning of multifaceted long-range relations among elements in data such as words (tokens) in sentences. The structural and algorithmic design empowers its scalability in order to utilize large-scale data. Currently, LCM efforts are all based on Transformer-like structures.

Research into LCMs is still in the early stages. Representative publications and preprints include scBERT [5], Geneformer [6], scGPT [7], scFoundation [8], tGPT [9], GeneCompass [10], scMulan [11] and UCE [13]. This mini-review provides an overview of these studies, focusing on their general framework, pretraining data, pretraining task design, cell and gene embeddings, and typical downstream tasks. It is too early to ask which is the best practice. We provide our perspectives regarding the open questions in this field.

## BASIC COMPONENTS OF SINGLE-CELL FOUNDATION MODELS

**Figure 1.**
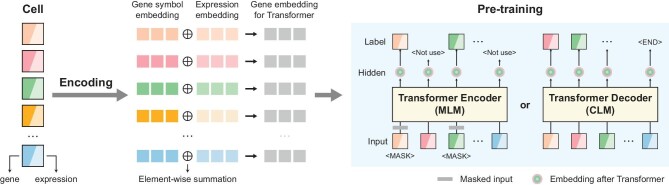
Overview of a typical Transformer-based LCM or foundation model for single-cell transcriptomics. Genes and their expression values are tokenized and encoded as input embeddings for the Transformer model. The model is pretrained for either a mask language modeling (MLM) task or a causal language modeling (CLM) task. The MLM pretraining task uses predefined mask tokens to replace some randomly chosen genes or expressions, and uses the truth genes and/or their expressions as the prediction targets. The CLM pretraining task makes the model predict the next gene in a predefined order, or all unseen genes and their expressions by training a Transformer Decoder. Typically, a prediction head is used to process the hidden outputs generated from the Transformer layers based on the labels.

Figure 1 shows the basic structure and information flow of typical Transformer-based LCMs. The gene expression data (and the extra metadata for scMulan) of each cell is taken as a training sample. The data first need to undergo a module to encode the genes as high-dimensional vectors called ‘embeddings’ to be input into the Transformer module. For building an LCM, one needs to collect and preprocess a large-scale single-cell transcriptomics data set, to design a method of tokenizing and encoding the data (see DATA TOKENIZATION AND ENCODING), to design a pretraining task on the data (see PRETRAINING TASK DESIGN) and a particular architecture of the Transformer modules (see TRANSFORMER STRUCTURE DESIGN). Different LCMs have different designs with regard to these key aspects. The studies used a variety of downstream tasks to show the utility and performance of the models (Table 1). Some of the tasks are common to all models, and some are more model-specific (see APPLYING SINGLE-CELL FOUNDATION MODELS TO DOWNSTREAM TASKS).

**Table 1.** Typical ways of applying single-cell foundation models. The table includes three major types of applications for single-cell foundation models categorized by the type of output information: gene-embedding applications for tasks such as gene property prediction and gene network inference; cell-embedding applications to analyze cell properties and similarities, supporting tasks such as batch correction, clustering, cell-type annotation and multi-omics integration; and applications of reconstructed or generated expression profiles, enabling tasks such as data augmentation, perturbation prediction and cross-modal prediction.

Application type	Downstream task	Models
Gene-embedding based	Gene network inference	[6,7,10]
	Gene property prediction	[6,7,10]
	Perturbation prediction	[6–8,10]
Cell-embedding based	Batch correction	[7,11]
	Cell clustering	[6–11,13]
	Cell type annotation	[6–8,10,11,13]
	Multi-omics integration	[7]
Profile based	Profile generation	[7,11]
	Data augmentation	[7,8]
	Cross-modal prediction	[34]

The models consider each cell as a sentence. The pretraining is based on a large number of such cell sentences of diverse cell types, tissues and donor statuses. The size of data sets has grown from millions of cells (e.g. scBERT) and tens of millions (e.g. scGPT, scFoundation, Geneformer, tGPT, scMulan and UCE), to more than 100 million (e.g. GeneCompass and CellFM [14]). Most studies only used human single-cell transcriptomic data. tGPT also included bulk RNA sequencing data and some single cell ATAC-seq data. GeneCompass and UCE incorporated data from mice and other species. Cell atlases like hECA, CZ-CELLxGENE and DISCO [15] are typical sources of data. scGPT and UCE used CZ-CELLxGENE, while scFoundation used HCA, DISCO, hECA and other resources. scMulan further leveraged the systematically organized and standardized metadata from hECA for additional information.

Data collected from different studies and sources need to undergo gene symbol unification and quality control. Gene symbol unification standardizes gene lists from different sources, providing a consistent gene expression space. Some models used highly variable genes (e.g. scMulan) and subsets of genes (e.g. tGPT), and some used all available protein-coding genes (e.g. scFoundation). The processed gene expression matrix is typically preprocessed with steps like normalization and log transformation.

## DATA TOKENIZATION AND ENCODING

The Transformer was invented for serialized data like natural languages. But single-cell transcriptomics data are of a tabular type, composed of genes and their expression values without any natural order. Therefore, we need to design a way to arrange the transcriptomics data so that they can be processed by the Transformer model.

In NLP, the basic unit of input is ‘token’, which could be a word, a sub-word or a punctuation mark. Tokens in sentences are obtained through a process called tokenization, which segments the sentence into tokens from a vocabulary. All tokens in a sentence are projected into high-dimensional vectors called representation embeddings, which together form the input to the Transformer. In transcriptomics, the genes (represented by their names) are the natural basic units and can be considered tokens. A cell can be represented as a ‘cell sentence’ composed of expressed gene tokens. However, unlike sentences in natural language, in each cell sentence, each gene is not only represented by its name token, but also has its expression value. It is necessary to separately encode the gene name (identity) and expression value into embeddings that can be processed by the Transformer. Typically, we can transform genes and their expression values into embedding spaces of the same dimensionality, and then obtain the final input embedding to the Transformer by element-wise summation of these two embeddings. There can be different ways to encode the gene names and the corresponding expression values.

### Gene name encoding

For gene name encoding, most models adopt the same method of token encoding used in NLP, where each gene is projected into a high-dimensional embedding space by one-hot encoding with a projection neural network. The projection process has learnable parameters that are updated during the training process, enabling the model to capture the relationships and differences between genes.

In addition to learning the embedding directly from the training data, GeneCompass assigned other embeddings to genes by introducing external knowledge, including promoter embeddings, co-expression embeddings, gene family embeddings and gene regulatory network embeddings. This additional information on DNA sequences and co-expression patterns enriched the content of the gene embeddings. These embeddings are aggregated to compose the input to the Transformer. UCE used the gene's protein product to encode the gene name. It used the pretrained protein language model ESM2 [16] to encode the protein product of a gene and take the average of all proteins coded by a gene as the embedding of the gene name. This design better accommodated a broader range of gene sets across species, allowing the model to generalize to other species.

Some studies also tried to encode gene names with the textual embeddings from LLMs, such as scELMo [17] and scInterpreter [18]. These models used scientific information on the gene from databases like NCBI [19] as a prompt, and got the embeddings from an LLM. These textual embeddings were regarded as the gene-name embedding, which may contain complex biological information about the gene.

### Gene expression values encoding

Currently there are four major encoding methods used to encode gene expression values into embedding space: rank encoding, continuous value encoding, discrete value encoding and reference encoding. These methods encode the information about gene expression values and add these encoded embeddings to the gene-name embeddings.

Rank encoding: Genes expressed in the cell can be sorted in descending order according to their expression, thus forming a gene sequence. The positions of genes are then indicated by positional encoding similar to that in NLP, forming an embedding that contains information about the relative level of expression. Geneformer and tGPT employed this strategy. This way of encoding gene expression easily fits the original Transformer encoding process, but the original expression values cannot be recovered by the model.

Continuous value encoding: The gene expression is a continuous value after preprocessing. We can map the value to the same space as the gene-name embedding with a projection neural network. scFoundation and GeneCompass used this strategy. It preserves the original continuous expression information at the price of bringing extra variability and complexity to the model.

Discrete value encoding: Continuous gene expression values can be first discretized into multiple bins. Each expression value then becomes a one-hot vector and therefore can be projected into the embedding space in the same way gene names are encoded. scGPT, scMulan and BioFormers [20] all used this strategy but with different ways of dividing the bins.

Reference encoding: The reference encoding method encodes expression value as a reference for the gene-name embeddings. For example, scEMLo used the expression value as the weight of gene-name embedding; UCE derived the probability of sampling a gene based on its expression value, and randomly selected a certain percentage of genes base on their probabilities.

### Encoding extra information

In addition to gene names and expression values, the extra information in the metadata can also be encoded into the inputs of the Transformer. The extra information can be important for the characterization of the cells. scMulan used this strategy to enable the model to capture relationships between gene expression and cell characters. It also included downstream task tokens to enable the model to perform zero-shot functions according to the task tokens. Other models used tokens such as batch tokens, classify tokens (CLS) and perturbation tokens. These tokens are encoded in the same way as gene name encoding, and are added into the gene name vocabularies. The embeddings of these special tokens can give additional information to the model.

Different LCMs used different encoding methods for genes, expression values and other information. The specific contributions of these encoding designs in foundation models have not yet been systematically studied [21–23]. It is still for the community to determine the best practice for turning genes and their expressions into vectors that AI models can work on.

## PRETRAINING TASK DESIGN

Model pretraining aims to train the model using data, by constructing self-supervised tasks without relying on supervision for specific downstream tasks. This allows the model to learn data distribution and interrelations to achieve efficient transfer capabilities. In NLP, common pretraining tasks include predicting randomly masked words in a sentence or predicting the next word based on preceding words. These tasks were used in BERT [24]-style models and GPT [25]-style models, respectively. Current pretraining tasks in single-cell foundation models also fall into two categories: masked language modeling (MLM) tasks similar to BERT-style pretraining, and causal language modeling (CLM) tasks similar to GPT-style pretraining (Fig. 1).

### Pretraining with masked language modeling

MLM is a common self-supervised pretraining method, typified by BERT and its variants in natural language processing [24,26–28]. Currently, most single-cell foundational models such as scBERT, Geneformer, scGPT and scFoundation use this pretraining task. In the MLM task, the gene names and their expressions in a cell are randomly masked, and the model is trained to predict masked genes and/or expression levels. scFoundation added a read-depth recovery task beyond the basic MLM task, which harmonizes the read-depth difference across data sets.

In the pretraining with MLM task, the Transformer first generates high-dimensional representations for each gene. Then the representations of the masked positions are used to predict expression levels or gene names. The pretrained model captures the complex structures and dependencies in the cell, which is crucial for understanding gene expression patterns and cell states. One limitation of this approach is that it is not well-suited for integrating extra information such as cell types or tissue sources in the metadata. The MLM task can also be sensitive to the masking strategy.

### Pretraining with causal language modeling

Advanced language models in natural language processing such as GPTs and Llama [25,29–31] utilize CLM as generative pretraining tasks. The CLM task involves predicting the next element given an input sequence, enabling task completion through generation during inference. Since single-cell gene expression lacks a natural order, different foundation models propose different variants of CLM tasks.

tGPT formatted the gene order based on expression levels, defining the pretraining task as predicting the next gene given the preceding gene order, which is to sequentially predict lower-expressed genes from higher-expressed ones. scMulan randomly shuffled genes within cells to eliminate the order information. It defined the pretraining task as predicting the remaining genes and their expression values given seen genes. Besides, scMulan included metadata such as cell type, organ name, donor age and donor gender into pretraining. By assigning metadata terms to different positions of the ‘cell sentences’, scMulan formatted different tasks establishing connections between gene expression and metadata. By setting task prompts, scMulan can generate corresponding contents for different tasks, enabling multitask pretraining with a unified paradigm.

The benefit of CLM pretraining is that it equips the foundation model with the ability to generate gene profiles and metadata, making it adaptable to a wide range of tasks, and also enabling zero-shot capabilities. However, a drawback is the need for large data sets that combine gene profiles with labeled metadata. Additionally, generative models trained with the CLM task do not explicitly obtain representations for each input gene from a single-cell expression profile, as the representation only contains information about this gene and its preceding genes. How to effectively extract gene representations remains an area for further research.

In both MLM and CLM pretraining, the loss is computed at the output end of the Transformer. The famous backpropagation (BP) algorithm is used to send gradient information back through all layers of the Transformer to iteratively update all learnable parameters. This is the phase that consumes most of the computing in foundation models.

## TRANSFORMER STRUCTURE DESIGN

The original Transformer architecture for language translation has two main components: an Encoder and a Decoder. Later foundational models usually use only one of these components according to the specific pretraining tasks. The models trained with MLM, such as scBERT, Geneformer, scGPT and scFoundation, only use the Transformer Encoder. scBERT and scFoundation apply a variant of the Transformer, the Performer [32], which has lower memory usage during pretraining. Models trained with CLM, including tGPT and scMulan, only use the Transformer Decoder.

The main difference between the Transformer Encoder and Decoder is in their attention mechanisms. The Encoder processes the entire input sequence simultaneously, using a bidirectional attention mechanism that allows each element to attend to all other elements in the sequence. This enables the model to capture global context and dependencies within the data. In MLM tasks, the model is trained to predict the masked genes based on the contextual genes, allowing the bidirectional attention mechanism to help the model fill in missing genes using global information.

In contrast, the Decoder processes the input sequence using a causal (unidirectional) attention mechanism. Each element can only attend to itself and the preceding elements, which is essential for generative tasks to generate the subsequent elements conditional on the previous information. In CLM tasks, the model is trained to predict subsequent genes or metadata terms, making the causal attention mechanism of the Transformer Decoder well-suited for this purpose.

Aside from the attention mechanisms, other components of the Encoder and Decoder such as the feedforward neural network, layer normalization and activation functions, are similar. Both the Encoder and Decoder stacks consist of layers that include multi-head attention, followed by a feedforward neural network. The output of each sub-layer is passed through a layer normalization step. Activation functions such as ReLU are typically used in the feedforward network. These components collectively contribute to the model's ability to learn complex patterns and relationships in the data.

## APPLYING SINGLE-CELL FOUNDATION MODELS TO DOWNSTREAM TASKS

In contrast to task-specific approaches, pretrained single-cell foundation models show their versatility in various downstream tasks and report better performance. Most of them execute tasks by fine-tuning on different assignments. Single-cell foundation models conduct downstream tasks through gene embedding, cell embedding and cell generation (Table 1).

### Gene-embedding-based applications

Single-cell foundation models output gene embeddings that convey biological meaning after pretraining. Gene embeddings are a vector for each gene. They are categorized as data-independent or contextual embeddings. Data-independent embeddings encode gene names, and are fixed once the model is pretrained. Contextual gene embeddings are obtained by inputting single-cell data into the model and retrieving outputs from the last layers of Transformers. They capture cross-gene attentions from the Transformer, encoding a gene's information and relation with others in the input context. Data-independent embeddings capture the characteristics or relationship of genes without expression values, which are used to infer global gene regulatory networks (GRN) in scGPT. Contextual embeddings reflect the gene function in certain input samples, and can be used to predict sample-specific properties such as dosage sensitivity [6] or to build cell-type-specific GRNs.

### Cell-embedding-based applications

Single-cell foundation models output cell embeddings as representations of individual cells. These embeddings are achieved by pooling all contextual gene embeddings or using the embedding of a special token like CLS.

Cell embeddings from single-cell foundation models enable tasks on cell characteristics in a representation-learning manner. A basic downstream task is cell-type annotation. With fine-tuning for the annotation task, most models outperform traditional approaches. scMulan supports zero-shot cell-type annotation based on encoded cell-type information in pretraining. Drug response prediction is another key task. By using cell embeddings to replace gene expression representation of cells, scFoundation improved the accuracy and resolution of drug response prediction on both bulk and single-cell data.

Cell embeddings also characterize cellular heterogeneity and can improve clustering performance [8,9]. Similarities of cell embeddings can be used to evaluate the hierarchical organization of cell types [13], detect novel subtypes [13] and track cellular changes across different health statuses and treatments [6]. To minimize technical biases across batches, models like scGPT and Geneformer use adversarial learning in fine-tuning while UCE and scMulan present batch-removed embeddings without additional fine-tuning.

Cell embeddings also provide a new strategy for integrating multi-omics data. scGPT incorporated additional tokens for ATAC or protein omics, and used a fine-tuning strategy similar to the multi-batch integration task. Integrated cell embeddings across omics offer a multifaceted view of cellular states and regulatory mechanisms.

### Profile-based applications

The profiles generated from the reconstruction process in MLM-pretrained models or the conditional generation process in CLM models enable profile-based applications such as data augmentation, perturbation prediction and cross-modal prediction. For instance, scFoundation enhances read depth and improves cell-type classification. To predict the response to gene knockout, downregulation or overexpression, foundation models are fine-tuned on perturbation data [7] or combined with existing methods like GEARS [8,10,33]. In cross-modal prediction, models use one modality to predict another. For example, scTranslator [34] uses transcriptomics data to predict proteomics data.

CLM-pretrained models enable conditional generation tasks. For instance, scMulan can generate expression profiles conditioned on metadata and some gene expression. Such generated data can predict perturbation responses or present the cell information in a novel status without additional sample-collection cost.

Single-cell foundation models also show potential in spatial transcriptomics applications. For example, models like scGPT, SpaFormer [35], CellPLM [36] and NicheFormer [37] have been directly applied to spatial genomics data, demonstrating effective results in spatial expression imputation tasks. SpaFormer and CellPLM encode spatial coordinates of cells into the positional encoding in the model input.

## FUTURE PERSPECTIVES

Single-cell foundation models have demonstrated impressive performance on multiple downstream tasks based on general pretraining with massive single-cell transcriptomic data. This illustrates the possibility and feasibility of using large AI models to capture the underlying biology contained in complex biology data. However, this is just the beginning. Current practices are still preliminary, with many important technical and scientific questions still to be understood, both on the AI side and biology side.

The practices in NLP and multimedia fields have illustrated the power of increasing the size and quality of data, and increasing the size of the models, to achieve higher levels of intelligence. Current observations of large cellular models suggest this could also be true in the field of biology, but more solid evidence is still needed. We should be aware of the fundamental difference between AI for text and images and AI for biology. In AI for biology, what we expect AI to do is to dig into data that are too challenging for human experts to analyze, and to answer questions that human intelligence alone struggles to answer [38].

While the community is eager to find the best foundation models to solve their scientific problems, it is too early to know what the best practices are in many aspects of the technology. A series of standardized benchmarking data sets and tasks are essential to compare technologies and evaluate solutions, but they are largely lacking due to technical and biological reasons [21]. Benching data and tasks at different levels of difficulty has played a major role in the evolution and development of AI models in CV and NLP. This is also needed for developing AI models for biology. We therefore call for more systematic efforts with regard to building benchmarking data sets and tasks using all possible approaches. Recent developments in synthetic biology and organelle technology, as well as systematic experiments on model organisms, could make major contributions.

The future of single-cell foundation models will also involve more data modalities and enable multi-omics tasks. By integrating spatial transcriptomics, models can achieve a more comprehensive understanding of cellular mechanisms within specific niches. Integrating temporal data, such as RNA velocity, will enable models to learn dynamic state transitions. Incorporating chromatin accessibility data will aid in the learning of more explicit regulatory relationships. These approaches will enhance the power and applicability of single-cell foundation models, leading to more accurate and insightful biological interpretations. Foundation models would further extend applications in in-silico experiments such as gene perturbation for disease target discovery and rapid drug screening, which could revolutionize biomedical research, offering new insights and accelerating the development of therapeutic interventions.

This is an exciting beginning of a new era, the era of building AI foundation models to learn, understand and simulate the biology of cells and of life [39]. Current progress is promising. There are many challenging open questions. But many more breakthroughs can be expected.
